# Obesity Indexes and Total Mortality among Elderly Subjects at High Cardiovascular Risk: The PREDIMED Study

**DOI:** 10.1371/journal.pone.0103246

**Published:** 2014-07-29

**Authors:** Miguel A. Martínez-González, Ana García-Arellano, Estefanía Toledo, Maira Bes-Rastrollo, Mónica Bulló, Dolores Corella, Montserrat Fito, Emilio Ros, Rosa Maria Lamuela-Raventós, Javier Rekondo, Enrique Gómez-Gracia, Miquel Fiol, Jose Manuel Santos-Lozano, Lluis Serra-Majem, J. Alfredo Martínez, Sonia Eguaras, Guillermo Sáez-Tormo, Xavier Pintó, Ramon Estruch

**Affiliations:** 1 Centro de Investigación Biomédica en Red de Fisiopatología de la Obesidad y Nutrición, Instituto de Salud Carlos II, Madrid, Spain; 2 The PREDIMED (Prevención con Dieta Mediterránea) Network (RD 06/0045) Instituto de Salud Carlos III, Madrid, Spain; 3 Department of Preventive Medicine and Public Health, University of Navarra-Osasunbidea, Servicio Navarro de Salud, Pamplona, Spain; 4 Human Nutrition Department, Hospital Universitari Sant Joan, Institut d'Investigació Sanitaria Pere Virgili, Universitat Rovira i Virgili, Reus, Spain; 5 Department of Preventive Medicine, University of Valencia, Valencia, Spain; 6 Cardiovascular and Nutrition Research Group, Institut de Recerca Hospital del Mar, Barcelona, Spain; 7 Lipid Clinic, Department of Endocrinology and Nutrition, Institut d'Investigacions Biomèdiques August Pi i Sunyer, Hospital Clinic, University of Barcelona, Barcelona, Spain; 8 Department of Nutrition and Food Science, School of Pharmacy, Xarxa de Referència en Tecnologia dels Aliments, Instituto de Investigación en Nutrición y Seguridad Alimentaria, University of Barcelona, Barcelona, Spain; 9 Department of Cardiology, University Hospital of Alava, Vitoria, Spain; 10 Department of Preventive Medicine, University of Malaga, Malaga, Spain; 11 Institute of Health Sciences, University of Balearic Islands, and Hospital Son Espases, Palma de Mallorca, Spain; 12 Department of Family Medicine, Primary Care Division of Seville, San Pablo Health Center, Seville, Spain; 13 Department of Clinical Sciences, University of Las Palmas de Gran Canaria, Las Palmas, Spain; 14 Department of Nutrition and Food Sciences, Physiology and Toxicology, University of Navarra, Pamplona, Spain; 15 Department of Biochemistry and Molecular Biology-Service of Clinical Analysis- University of Valencia, Valencia, Spain; 16 Lipids and Vascular Risk Unit, Internal Medicine, Hospital Universitario de Bellvitge, Hospitalet de Llobregat, Barcelona, Spain; 17 Department of Internal Medicine Institut d'Investigacions Biomèdiques August Pi i Sunyer, Hospital Clinic, University of Barcelona, Barcelona, Spain; University of Valencia, Spain

## Abstract

**Background:**

Different indexes of regional adiposity have been proposed for identifying persons at higher risk of death. Studies specifically assessing these indexes in large cohorts are scarce. It would also be interesting to know whether a dietary intervention may counterbalance the adverse effects of adiposity on mortality.

**Methods:**

We assessed the association of four different anthropometric indexes (waist-to-height ratio (WHtR), waist circumference (WC), body mass index (BMI) and height) with all-cause mortality in 7447 participants at high cardiovascular risk from the PREDIMED trial. Forty three percent of them were men (55 to 80 years) and 57% were women (60 to 80 years). All of them were initially free of cardiovascular disease. The recruitment took place in 11 recruiting centers between 2003 and 2009.

**Results:**

After adjusting for age, sex, smoking, diabetes, hypertension, intervention group, family history of coronary heart disease, and leisure-time physical activity, WC and WHtR were found to be directly associated with a higher mortality after 4.8 years median follow-up. The multivariable-adjusted HRs for mortality of WHtR (cut-off points: 0.60, 0.65, 0.70) were 1.02 (0.78–1.34), 1.30 (0.97–1.75) and 1.55 (1.06–2.26). When we used WC (cut-off points: 100, 105 and 110 cm), the multivariable adjusted Hazard Ratios (HRs) for mortality were 1.18 (0.88–1.59), 1.02 (0.74–1.41) and 1.57 (1.19–2.08). In all analyses, BMI exhibited weaker associations with mortality than WC or WHtR. The direct association between WHtR and overall mortality was consistent within each of the three intervention arms of the trial.

**Conclusions:**

Our study adds further support to a stronger association of abdominal obesity than BMI with total mortality among elderly subjects at high risk of cardiovascular disease. We did not find evidence to support that the PREDIMED intervention was able to counterbalance the harmful effects of increased adiposity on total mortality.

**Trial Registration:**

Controlled-Trials.com ISRCTN35739639

## Introduction

The dose-response relationship between increased levels of overweight/obesity and all-cause mortality is controversial. A recent meta-analysis reported that overweight (defined as abody mass index [BMI] of 25 to 30 kg/m^2^) was associated with significantly *lower* all-cause mortality in comparison with the normal weight category and that grade I obesity (BMI between 30 and 35) was associated with a non-significantly *lower* risk of mortality [1]. Other previous meta-analyses also suggested inverse or null associations, specially among elderly subjects [2], [3]. In contrast with these reports, there is a high biological plausibility to expect a direct association between excess body weight and all-cause mortality even at moderate levels of overweight. Some potential biases may have attenuated the association. They include insufficient adjustment for confounding by smoking, reverse causation bias due to disease-related weight loss [4], effect modification by age [5], [6], with an attenuation of the detrimental effects of overweight or even a reversion to beneficial effects in the elderly [7]. In addition, studies using BMI as the single relevant measurement of adiposity and not other aspects of body composition such as visceral fat or fat distribution, may miss the true dose-response curve between adiposity and all-cause mortality. Alternative measures of abdominal obesity are known to be superior to BMI in showing a positive association with the risk of cardiovascular disease or diabetes [8], [9], [10], [11]. In addition, a recent study has clearly shown significant associations between waist circumference or other indexes of abdominal obesity and all-cause mortality in white subjects [12]. Furthermore, recent studies have suggested that the BMI should be refined by measuring additional indexes of fat distribution namely waist circumference (WC), waist-to-hip ratio (WHR) or waist-to height ratio (WHtR) to better identify higher-risk subjects [13], [14].

WC has been often used to appraise abdominal obesity, but WC does not take differences in height into account, and subjects with a given WC will have more abdominal fat than taller subjects with the same WC [15]. The WHtR represents a further step because it also takes height into account. A systematic review found mean areas under receiving operator characteristic curves of 0.704, 0.693 and 0.671 for WHtR, WC and BMI, respectively as predictors of diabetes or cardiovascular disease [10].

We assessed the association between several anthopometric indexes (BMI, WC, WHtR, Height) and all-cause mortality in a Mediterranean cohort of elderly subjects at high cardiovascular risk included in the PREDIMED trial. The primary objective of the PREDIMED trial was to test the effect of a Mediterranean-style diet in primary cardiovascula prevention.

## Methods

The protocol for the PREDIMED trial is available as supporting information at www.predimed.es. We have previously reported the design, objectives and methods of the PREDIMED trial in a specific publication [16]. The PREDIMED study is a multicenter, randomized, primary cardiovascular prevention trial conducted in Spain (www.predimed.es). The final results were reported in 2013 [17]. We randomly assigned participants to one of three diets: a Mediterranean diet (MeDiet) supplemented with extra-virgin olive oil, a MeDiet supplemented with mixed nuts, or a control group (allocated to receive advice to reduce dietary fat). All analyses were stratified by intervention group and adjusted for potential confounding.

During the analysis (with the exception of spline models, see below) we used the lowest group of each anthopometric index as the reference category.

The protocol of this study was approved by the Institutional Review Board of the University of Navarra and the Institutional Review Board of the Hospital Clinic (Barcelona). The Data Protection Agency is the Spanish National Agency (Agencia Española de Protección de Datos), name of the file: PREDIMED, the responsible person for the file is Miguel A. Martínez-González.

The trial is registered at http://www.controlled-trials.com/ISRCTN 35739639, and all clinical investigation have been conducted according to the principles expressed in the Declaration of Helsinki. Subjects who met entry requirements agreed to participate and provided written informed consent.

### Subjects

We enrolled 7,447 participants. Forty three percent of them were men (55 to 80 years) and 57% were women (60 to 80 years). All of them were initially free of cardiovascular disease, but at high cardiovascular risk because they had at least three major cardiovascular risk factors out of six candidate risk factors or, alternatively, they were type 2 diabetics. The 6 candidate risk factors considered were: overweight/obesity, hypertension, elevated low-density lipoprotein cholesterol, low high-density lipoprotein cholesterol, current smoking, or family history of premature coronary heart disease. The specific cut-off points for these factors and the exclusion criteria have been previously described [16]. Participants can be assumed to be on stable weights at the time of recruitment for the trial. Energy restriction was not part of the PREDIMED nutritional intervention.

The recruitment took place in 11 recruiting centers between 2003 and 2009. Eighty-nine percent of candidate subjects who met entry requirements agreed to participate and provided written informed consent.

### Measurements

Registered nurses who had been previously trained and certified to implement the PREDIMED protocol directly measured weight, height and WC of participants as previously described [16], [18], [19]. Height (m) and weight (kg) were measured with light clothing and no shoes with calibrated scales and a wall-mounted stadiometer, respectively; BMI was calculated as the weight in kilograms divided by the square of the height in meters; WC was measured midway between the lowest rib and the iliac crest using an anthropometric tape; in other previous studies, the protocol for WC measurement specified that WC should be measured at the minimal waist (33%), midpoint (26%) and umbilicus (27%). We selected the midpoint. However, the available evidence from a 2008 meta-analysis suggests that WC measurement protocol has no substantial influence on the association between WC, all-cause and CVD mortality, CVD and diabetes [20]; the WHtR was calculated as WC divided by height, both in centimeters. Blood pressure was measured in triplicate using a validated semiautomatic oscillometer with a 5-minute interval between each measurement and the subject in a sitting position (Omron HEM-705CP, Hoofddorp, The Netherlands). Hypertension was defined as a systolic blood pressure ≥140 mm Hg, a diastolic blood pressure ≥90 mm Hg, or the use of antihypertensive therapy.

### Confounders assessment

Participants underwent a baseline interview that included the evaluation of cardiovascular risk factors and physician diagnoses of hypertension, diabetes and hipercolesterolemia. At the same time we gathered information about medical, socio-demographic, anthropometric, and lifestyle variables. We used the Minnesota validated physical activity questionnaire to assess leisure-time physical activity [21], [22], Time spent in several activities in minutes per day was multiplied by its typical energy expenditure, expressed in metabolic equivalent tasks (METs), then summed over all activities to yield a METs-min/d score for each participant. Taking into account that the relationship between leisure-time physical activity (METs-min/d) and mortality was not linear in our data, we used a polynomial model to adjust for METs-min/d, adding a quadratic term to the multivariable model. The age range in the validation studies was 18–60 years. Dietary habits were collected through a semi-quantitative 137-item Food Frequency Questionnaire previously validated in Spain [23].

### Statistical analysis

We examined baseline characteristics of participants in each variable of interest according to quartiles or to predefined categories of anthropometric indexes. To compare means or percentages of each variable across quartiles (or pre-defined categories) of anthropometric indexes we used one-way ANOVA and chi-squared tests, respectively.

We used Cox regression models to assess the Hazard Ratios (HR) and their 95% confidence intervals for total mortality according to quartiles or to pre-defined categories of each anthropometric index.

For the multiple-adjusted model, the following potential confounders (all of them measured at baseline) were considered: age, sex, smoking, diabetes status, hypertensive status, intervention group and family history of CHD.

To assess the dose-response shape between adiposity and mortality we used restricted cubic splines models with 4 degrees of freedom, and considered the point associated with the lowest mortality as the reference value for each anthropometric index.

Finally, we also obtained the hazard ratios and their 95% confidence intervals for total mortality according to defined cut-off points of each anthropometric index. A p value <0.05 was considered statistically significant.

Analyses were performed using STATA version 12.1 (StataCorp, College Station, TX, USA).

## Results

Table 1 shows baseline characteristics of the 7447 participants of PREDIMED according to quartiles (or pre-defined categories) of anthropometric indexes. We studied baseline characteristics by four variables of interest: WHtR, WC, BMI and height. Baseline risk factors (hypertension, overweight, type-2-diabetes) increased across increasing quartiles, as expected, with the exceptions of dyslipidemia and family history of premature CHD, that decreased with increasing quartiles. We also observed that adherence to the MeDiet tended to decrease across increasing quartiles of anthropometric indexes. Leisure-time physical activity followed the same inverse trend across adiposity indexes, but not across height quartiles, where a *direct* association was apparent. There were other differences in variables such as smoking or hypertension but they did not follow a consistent pattern across successive quartiles.

**Table 1:** Baseline Characteristics of Participants according to categories of waist-to-height ratio, waist circumference, body mass index and height.

Waist to height Ratio (WHtR)			
Characteristic	Lowest quartile N = 1865	Quartiles 2–3 N = 3734	Highest quartile N = 1848
Waist-to-height ratio (mean ± SD)	0.55±0.03	0.63±0.02	0.71±0.04
Waist circumference – cm (mean ± SD)	89±7.3	101±6.2	111±8.2
Body mass index (mean ± SD)†	26.4±2.5	29.9±2.8	33.7±3.3
Female sex – (%)†	51.3	53.8	71.2
Age-, – yr (mean ± SD)†	66.0±6.1	67.0±6.2	67.9±6.1
Smoking – (%)†			
Never	56.8	58.5	71.3
Former smoker	25.6	26.8	19.4
Current	17.6	14.7	9.3
Overweight (BMI ≥25) – (%)†	75.4	97.4	99.9
Obesity (BMI ≥30) – (%)†	7.2	46.0	89.2
Hypertension – (%)†	77.4	83.3	87.1
Type-2 diabetes – (%)†	45.3	48.3	52.4
Dyslipidemia – (%)	73.4	71.8	72.2
Family history of premature CHD – (%)†	26.1	21.3	20.8
Leisure-time physical activity (METS min/d) (mean ± SD)†	263.3±252.6	242.2±248.2	173.5±193.4
Intervention group – (%)†			
MeDiet+EVOO	33.6	34.7	33.6
MeDiet+nuts	36.0	32.9	30.0
Control	30.4	32.4	36.4
MeDiet Adherence score (mean ± SD)†	9.0±2.0	8.6±2.0	8.3±2.0

Waist Circumference (WC)			
Characteristic	Lowest quartileN = 2029	Quartiles 2–3N = 3661	Highest quartileN = 1757
Waist circumference – cm (mean ± SD)	88±5.6	101±3.6	114±6.5
Waist-to-height ratio (mean ± SD)	0.56±0.44	0.63±0.38	0.70±0.54
Body mass index (mean ± SD)†	27.0±2.9	29.9±3.0	33.4±3.5
Female sex – (%)†	75.3	53.5	45.4
Age-, – yr (mean ± SD)	67.1±6.0	67.0±6.3	66.7±6.3
Smoking – (%)†			
Never	71.8	59.3	53.3
Former smoker	16.2	26.4	30.8
Current	12.1	14.3	15.9
Overweight (BMI ≥25) – (%)†	78.2	97.0	99.7
Obesity (BMI ≥30) – (%)†	14.0	46.4	86.3
Hypertension – (%)†	79.8	82.9	85.8
Type-2 diabetes – (%)†	43.7	49.3	52.4
Dyslipidemia – (%)†	75.7	72.0	68.9
Family history of premature CHD – (%)†	25.9	21.9	19.4
Leisure-time physical activity (METS min/d) (mean ± SD) (mean ± SD)†	230.3±223.8	244.1±249.9	201.4±231.5
Intervention group – (%)			
MeDiet+EVOO	34.8	34.2	33.4
MeDiet+nuts	33.9	32.9	31.9
Control	31.4	32.9	34.7
MeDiet Adherence score (mean ± SD)†	8.9±2.0	8.6±2.1	8.4±2.0

Body Mass Index (BMI)			
Characteristics	<25 kg/m^2^ N = 563	>25 & <30 kg/m^2^ N = 3402	>30 kg/m^2^ N = 3482
Body mass index (mean ± SD)	23.5±1.2	27.7±1.4	33.2±2.7
Waist-to-height ratio (mean ± SD)†	0.54±0.05	0.60±0.05	0.67±0.06
Waist circumference – cm (mean ± SD)†	88±8.1	96±8.2	106±9.1
Female sex – (%)†	55.1	51.8	63.4
Age-, – yr (mean ± SD) |	67.4±6.4	67.0±6.3	66.9±6.1
Smoking – (%)†			
Never	56.0	57.3	66.1
Former smoker	24.3	26.8	22.6
Current	19.7	16.0	11.3
Hypertension – (%)†	72.3	80.5	86.7
Type-2 diabetes – (%)†	64.5	46.2	48.2
Dyslipidemia – (%)	73.8	72.8	71.7
Family history of premature CHD – (%)†	30.9	21.4	22.0
Leisure-time physical activity (METS min/d) (mean ± SD) (mean ± SD)†	258.5±265.5	260.6±256.2	196.3±211.7
Intervention group – (%)|			
MeDiet+EVOO	34.6	33.9	34.3
MeDiet+nuts	36.2	34.2	31.2
Control	29.1	31.9	34.5
MeDiet Adherence score (mean ± SD)†	8.8±2.0	8.8±2.0	8.4±2.0

Height			
Characteristics according to height.	Lowest quartile N = 1960	Quartiles 2–3 N = 3859	Highest quartile N = 1628
Height – m (mean ± SD)	1.49±0.03	1.60±0.04	1.73±0.04
Waist-to-height ratio (mean ± SD)†	0.65±0.07	0.63±0.06	0.61±0.06
Waist circumference – cm (mean ± SD)†	97±10.3	100±10.5	105±9.7
Body mass index (mean ± SD)†	30.8±4.1	29.9±3.8	29.2±3.4
Female sex – (%)†	97.1	59.5	5.0
Age-, – yr (mean ± SD)†	66.9±5.9	67.0±6.0	64.9±6.5
Smoking – (%)†			
Never	87.9	62.1	27.3
Former smoker	7.5	24.1	46.8
Current	4.6	13.8	25.9
Overweight (BMI ≥25) – (%)†	95.1	91.9	90.9
Obesity (BMI ≥30) – (%)†	55.2	46.2	39.1
Hypertension – (%)†	86.2	82.6	78.9
Type-2 diabetes – (%)†	46.5	48.6	50.8
Dyslipidemia – (%)|	76.4	72.3	67.2
Family history of premature CHD – (%)|	24.4	22.4	19.9
Leisure-time physical activity (METS min/d) (mean ± SD) (mean ± SD)†	176.9±171.6	231.7±233.9	291.6±299.4
Intervention group – (%)|			
MeDiet+EVOO	33.8	34.3	34.3
MeDiet+nuts	30.3	33.3	32.4
Control	34.3	35.3	30.4
MeDiet Adherence score (mean ± SD)†	8.4±2.0	8.6±2.0	8.8±2.1

† : p<0.001.

| : p<0.05.

The relationship between quartiles of anthropometric indexes and the risk of all-cause mortality is reflected in Table 2. We observed that the hazard ratio tended to be highest in the top quartiles for all anthropometric indexes.

**Table 2:** Hazard Ratios (95% confidence intervals) for total mortality according to quartiles of the waist-to-height ratio, waist circumference, height and categories of body mass index.

	Quartiles of waist-to-height ratio				
HR according waist-to-height-ratio	1 (lowest)	2	3	4 (highest)	
Limits	0.30 to 0.59	0.59 to 0.63	0.63 to 0.67	0.67 to 1.00	
Number of deaths	83	75	89	101	
Person-years	8188	8132	8059	7642	**P for trend**
Age-, sex-adjusted HR	1 (ref.)	**0.88** (0.64–1.20)	**1.01** (0.75–1.37)	**1.39** (1.02–1.88)	0.027
Multivariable adjusted^1^	1 (ref.)	**0.98** (0.72–1.35)	**1.01** (0.74–1.38)	**1.44** (1.05–1.97)	0.026
**Men**					
Number of deaths	60	51	54	49	
Person-years	3861	4022	3498	2185	
Age-adjusted HR	1 (ref.)	**0.80** (0.55–1.16)	**0.85** (0.58–1.22)	**1.28** (0.87–1.88)	0.344
Multivariable adjusted^1^	1 (ref.)	**0.85** (0.58–1.24)	**0.76** (0.52–1.12)	**1.31** (0.87–1.97)	0.430
**Women**					
Number of deaths	23	24	35	52	
Person-years	4327	4110	4561	5457	
Age-adjusted HR	1 (ref.)	**1.07** (0.59–1.94)	**1.42** (0.81–2.51)	**1.65** (0.94–2.88)	0.045
Multivariable adjusted^1^	1 (ref.)	**1.14** (0.63–2.05)	**1.38** (0.78–2.45)	**1.78** (1.02–3.11)	0.026

	Quartiles of waist circumference				
HR according waist circumference	1 (lowest)	2	3	4 (highest)	
Limits	50 to 94	94.3 to 100	100.6 to 107	107.5 to 177	
Number of deaths	75	70	97	106	
Person-years	9107	7476	8168	7271	**P for trend**
Age-, sex-adjusted HR	1 (ref.)	**1.01** (0.72–1.42)	**1.17** (0.85–1.61)	**1.42** (1.03–1.96)	0.020
Multivariable adjusted^1^	1 (ref.)	**1.03** (0.73–1.46)	**1.18** (0.86–1.63)	**1.36** (0.98–1.90)	0.046
**Men**					
Number of deaths	36	38	66	74	
Person-years	2185	3158	4179	4045	
Age-adjusted HR	1 (ref.)	**0.73** (0.46–1.15)	**0.92** (0.61–1.38)	**1.07** (0.72–1.61)	0.323
Multivariable adjusted^1^	1 (ref.)	**0.74** (0.47–1.19)	**0.88** (0.58–1.34)	**1.04** (0.67–1.61)	0.461
**Women**					
Number of deaths	39	32	31	32	
Person-years	6921	4318	3989	3226	
Age-adjusted HR	1 (ref.)	**1.42** (0.87–2.30)	**1.43** (0.86–2.40)	**1.88** (1.13–3.15)	0.018
Multivariable adjusted^1^	1 (ref.)	**1.42** (0.87–2.30)	**1.40** (0.84–2.34)	**2.02** (1.21–3.38)	0.012

	Quartiles of Body mass index				
HR according body mass index	1 (lowest)	2	3	4 (highest)	
Limits	17.78 to 27.22	27.23 to 29.73	29.74 to 32.45	32.46 to 52.97	
Number of deaths	98	78	82	90	
Person-years	9033	8002	8083	7902	**P for trend**
Age-, sex-adjusted HR	1 (ref.)	**0.80** (0.60–1.08)	**0.92** (0.68–1.24)	**1.22** (0.91–1.65)	0.159
Multivariable adjusted^1^	1 (ref.)	**0.80** (0.59–1.08)	**0.84** (0.63–1.14)	**1.14** (0.85–1.53)	0.371
**Men**					
Number of deaths	67	55	48	44	
Person-years	3756	3950	3567	2293	
Age-, sex-adjusted HR	1 (ref.)	**0.80** (0.56–1.14)	**0.83** (0.57–1.20)	**1.24** (0.85–1.83)	0.451
Multivariable adjusted^1^	1 (ref.)	**0.72** (0.50–1.04)	**0.67** (0.46–0.99)	**1.05** (0.70–1.56)	0.858
**Women**					
Number of deaths	31	23	34	46	
Person-years	4277	4052	4516	5609	
Age-adjusted HR	1 (ref.)	**0.80** (0.47–1.36)	**1.06** (0.64–1.73)	**1.22** (0.77–1.95)	0.245
Multivariable adjusted^1^	1 (ref.)	**0.79** (0.45–1.40)	**1.04** (0.64–1.69)	**1.22** (0.74–2.00)	0.264

	Quartiles of height				
HR according height	1 (lowest)	2	3	4 (highest)	
Limits (cm)	135 to 153	154 to 159	160 to 167	168 to 191	
Number of deaths	70	64	109	105	
Person-years	8518	7746	8947	6810	**P for trend**
Age-, sex-adjusted HR	1 (ref.)	**0.99** (0.70–1.41)	**1.12** (0.75–1.65)	**1.32** (0.86–2.04)	0.096
Multivariable adjusted^1^	1 (ref.)	**0.99** (0.70–1.41)	**1.05** (0.71–1.54)	**1.18** (0.76–1.83)	0.380
**Men**					
Limits (cm)	142 to 163	164 to 167	168 to 172	173 to 191	
Number of deaths	61	49	56	48	
Person-years	3883	3185	3711	2787	
Age-adjusted HR	1 (ref.)	**1.14** (0.79–1.66)	**1.16** (0.81–1.66)	**1.52** (1.04–2.22)	0.046
Multivariable adjusted^1^	1 (ref.)	**1.06** (0.73–1.53)	**1.03** (0.72–1.48)	**1.35** (0.91–2.02)	0.213
**Women**					
Limits (cm)	135 to 150	151 to 154	155 to 159	160 to 178	
Number of deaths	39	33	38	24	
Person-years	4767	4797	5213	3678	
Age-adjusted HR	1 (ref.)	**0.95** (0.60–1.52)	**1.13** (0.71–1.79)	**1.20** (0.71–2.06)	0.430
Multivariable adjusted^1^	1 (ref.)	**0.83** (0.52–1.34)	**1.02** (0.64–1.65)	**1.03** (0.59–1.81)	0.790

The PREDIMED study 2003–2010.

1 Adjusted for age, (sex, when pertinent) smoking, diabetes status, hypertensive status, intervention group and family history of CHD.

All estimates are stratified for study center. The interaction term between age and each antropometric index was not statistically significant in all analyses: p = 0.34 (for waist-to- height ratio); p = 0.53 (for waist circumference); p = 0.35 (for height) and p = 0.77 (for body mass index).

For WHtR, the hazard ratios slightly and non-significantly decreased in the second quartile and then increased in the upper quartiles. Thus, the age- and sex-adjusted HRs (95% confidence intervals) for categories of low, moderate, and high/very high WHtR compared to the lowest (reference) category were 0.88 (0.64–1.20), 1.01 (0.75–1.37) and 1.39 (1.02–1.88), respectively with a statistically significant linear trend (p = 0.027). In multivariable-adjusted models, the HRs were 0.98 (0.72–1.35), 1.01 (0.74–1.38), and 1.44 (1.05–1.97) for categories of low, moderate, and high/very high WHtR, respectively, with a significant linear trend (p = 0.026). Although the p for interaction with sex was not statistically significant (p for interaction WHtR × sex  = 0.34), when we separated men and women, no significant association was observed for men. But among women, a significant linear trend remained apparent (p = 0.045 and p = 0.026 for age-adjusted, and multivariable-adjusted models, respectively).

We observed that the hazard ratios for WC also increased across succesive quartiles. The age- and sex-adjusted HRs (95% confidence intervals) for quartiles of low, moderate, and high/very high WC compared to the lowest quartile (ref.) were 1.01 (0.72–1.42), 1.17 (0.85–1.61) and 1.42 (1.03–1.96) respectively. The linear trend was statistically significant (p = 0.020). After multivariable adjustment, the HRs were 1.03 (0.73–1.46), 1.18 (0.86–1.63) and 1.36 (0.98–1.90) with a significant linear trend (p = 0.046). Although the p for interaction with sex was not statistically significant (p for interaction WC × sex  = 0.53), when we separated men and women, the age-adjusted HRs were not significant among men. Among women the age -adjusted HRs were 1.42 (0.87–2.30), 1.43 (0.86–2.40) and 1.88 (1.13–3.15), respectively. After multivariable-adjustment, these HRs were 1.42 (0.87–2.30), 1.40 (0.84–2.34) and 2.02 (1.21–3.38). The linear trend among women was significant in both age-adjusted and multivariable-adjusted models (p = 0.018 and 0.012 respectively).

For BMI the HRs increased across successive quartiles although the results were not significant. The age- and sex-adjusted HRs (95% confidence intervals) for quartiles of low, moderate, and high/very high BMI compared to the lowest quartile (ref.) were 0.80 (0.60–1.08), 0.92 (0.68–1.24) and 1.22 (0.91– 1.65). After multivariable adjustment, these HRs were 0.80 (0.59–1.08), 0.84 (0.63–1.14) and 1.14 (0.85–1.53). The linear trend was not significant in any of both cases. The interaction with sex was not significant (p = 0.77). When we separated men and women, no significant association was observed for either category although among women the association was stronger.

When we classified the sample according to quartiles of height we also observed increased hazard ratios across successive quartiles. The p for interaction with sex was not significant (p = 0.35). When we studied each sex separately we observed that the HRs increased in both cases across successive quartiles. However among men this increase was higher, although in both cases, after multivariable adjustment, we observed no significant association between height and total mortality.

We conducted similar analyses using *a priori* defined cut-off points instead of quartiles. We observed in Table 3 that hazard ratios tended to be highest in the upper categories of the anthropometric indexes, in the same way as we observed in Table 2. We observed also that the hazard ratio decreased in the second category and then it increased in the upper categories.

**Table 3:** Hazard Ratios (95% confidence intervals) for total mortality according to categories (a priori defined cut-off points) of the waist-to-height ratio and waist circumference.

	Waist-to-height ratio				
Limits	< = 0.60	>0.60 to 0.65	>0.65 to 0.70	>0.70	
Number of deaths	104	106	87	51	
Person-years	10841	10485	6921	3774	**P for trend**
Age-, sex-adjusted HR	1 (ref.)	**1.01** (0.77–1.32)	**1.28** (0.96–1.71)	**1.58** (1.11–2.26)	0.009
Multivariable adjusted^1^	1 (ref.)	**1.02** (0.78–1.34)	**1.30** (0.97–1.75)	**1.55** (1.06–2.26)	0.013
**Men**					
Number of deaths	75	69	46	24	
Person-years	5230	4887	2613	837	
Age-adjusted HR	1 (ref.)	**0.91** (0.66–1.25)	**1.08** (0.74–1.56)	**1.62** (1.01–2.62)	0.148
Multivariable adjusted^1^	1 (ref.)	**0.82** (0.59–1.13)	**1.01** (0.69–1.49)	**1.59** (0.94–2.67)	0.238
**Women**					
Number of deaths	29	37	41	27	
Person-years	5611	5598	4308	2937	
Age-adjusted HR	1 (ref.)	**1.29** (0.78–2.13)	**1.70** (1.02–2.83)	**1.67** (0.94–2.97)	0.036
Multivariable adjusted^1^	1 (ref.)	**1.34** (0.81–2.22)	**1.82** (1.09–3.06)	**1.84** (1.03–3.29)	0.016

	Waist circumference				
**Limits (cm)**	**< = 100**	**>100 to 105**	**>105 to 110**	**>110**	
Number of deaths	119	72	57	100	
Person-years	13000	6632	5651	6737	**P for trend**
Age-adjusted HR	1(ref.)	**1.14** (0.85–1.53)	**1.04** (0.76–1.43)	**1.69** (1.29–2.23)	0.002
Multivariable adjusted^1^	1(ref.)	**1.18** (0.88–1.59)	**1.02** (0.74–1.41)	**1.57** (1.19–2.08)	0.008
**Men**					
**Limits**	**< = 100**	**>100 to 105**	**>105 to 110**	**>110**	
Number of deaths	68	45	36	65	
Person-years	4721	3108	2642	3096	
Age-adjusted HR	1 (ref.)	**1.01** (0.69–1.46)	**0.89** (0.59–1.33)	**1.43** (1.01–2.02)	0.093
Multivariable adjusted^1^	1 (ref.)	**0.98** (0.67–1.42)	**0.87** (0.58–1.31)	**1.32** (0.92–1.90)	0.204
**Women**					
**Limits**	**< = 95**	**>95 to 100**	**>100 to 105**	**>105**	
Number of deaths	40	28	18	48	
Person-years	6982	3558	3285	4630	
Age-adjusted HR	1 (ref.)	**1.45** (0.88–2.39)	**0.99** (0.55–1.80)	**1.93** (1.21–3.07)	0.012
Multivariable adjusted^1^	1 (ref.)	**1.40** (0.85–2.31)	**1.08** (0.60–1.94)	**1.93** (1.21–3.07)	0.012

	Height				
**Limits (cm)**	**< = 165**	**>165 to 170**	**>170 to 175**	**>175**	
Number of deaths	175	90	52	31	
Person-years	16303	8849	4854	2014	**P for trend**
Age-, sex-adjusted HR	1 (ref.)	**0.99** (0.77–1.28)	**1.10** (0.80–1.52)	**1.57** (1.07–2.31)	0.038
Multivariable adjusted^1^	1 (ref.)	**0.95** (0.73–1.23)	**1.05** (0.77–1.45)	**1.42** (0.97–2.08)	0.247
**Men**					
**Limits (cm)**	**< = 165**	**>165 to 170**	**>170 to1.75**	**>175**	
Number of deaths	91	56	39	28	
Person-years	5510	4012	2762	1282	
Age-adjusted HR	1 (ref.)	**0.96** (0.69–1.34)	**1.11** (0.76–1.62)	**1.73** (1.13–2.63)	0.046
Multivariable adjusted^1^	1 (ref.)	**0.89** (0.64–1.25)	**1.03** (0.70–1.52)	**1.57** (0.99–2.51)	0.213
**Women**					
**Limits (cm)**	**>155**	**>155 to 160**	**>160 to 1.65**	**>165**	
Number of deaths	84	34	13	3	
Person-years	10793	4837	2092	732	
Age-adjusted HR	1 (ref.)	**1.07** (0.71–1.60)	**1.11** (0.61–2.01)	**0.97** (0.29–3.22)	0.430
Multivariable adjusted^1^	1 (ref.)	**1.06** (0.69–1.61)	**0.97** (0.51–1.87)	**1.04** (0.33–3.26)	0.790

The PREDIMED study 2003–2010.

1 Adjusted for age, smoking, diabetes status, hypertensive status, intervention group and family history of CHD.

All estimates are stratified for study center. The interaction term was not statistically significant in case of waist-to-height ratio (p = 0.45).

When the analyses were restricted to deaths occurring after > = 2 yr follow-up, the only noticeable change was for the category >0.65 to 0.70 which exhibited a higher and significant elevated risk of death in the overall sample (multivariable-adjusted HR = 1.43, 95% CI: 1.03–1.97 in the total sample; 1.14 (0.77–2.42) in men, and 1.88 (1.09–3.24) in women).

**Figure 1:**
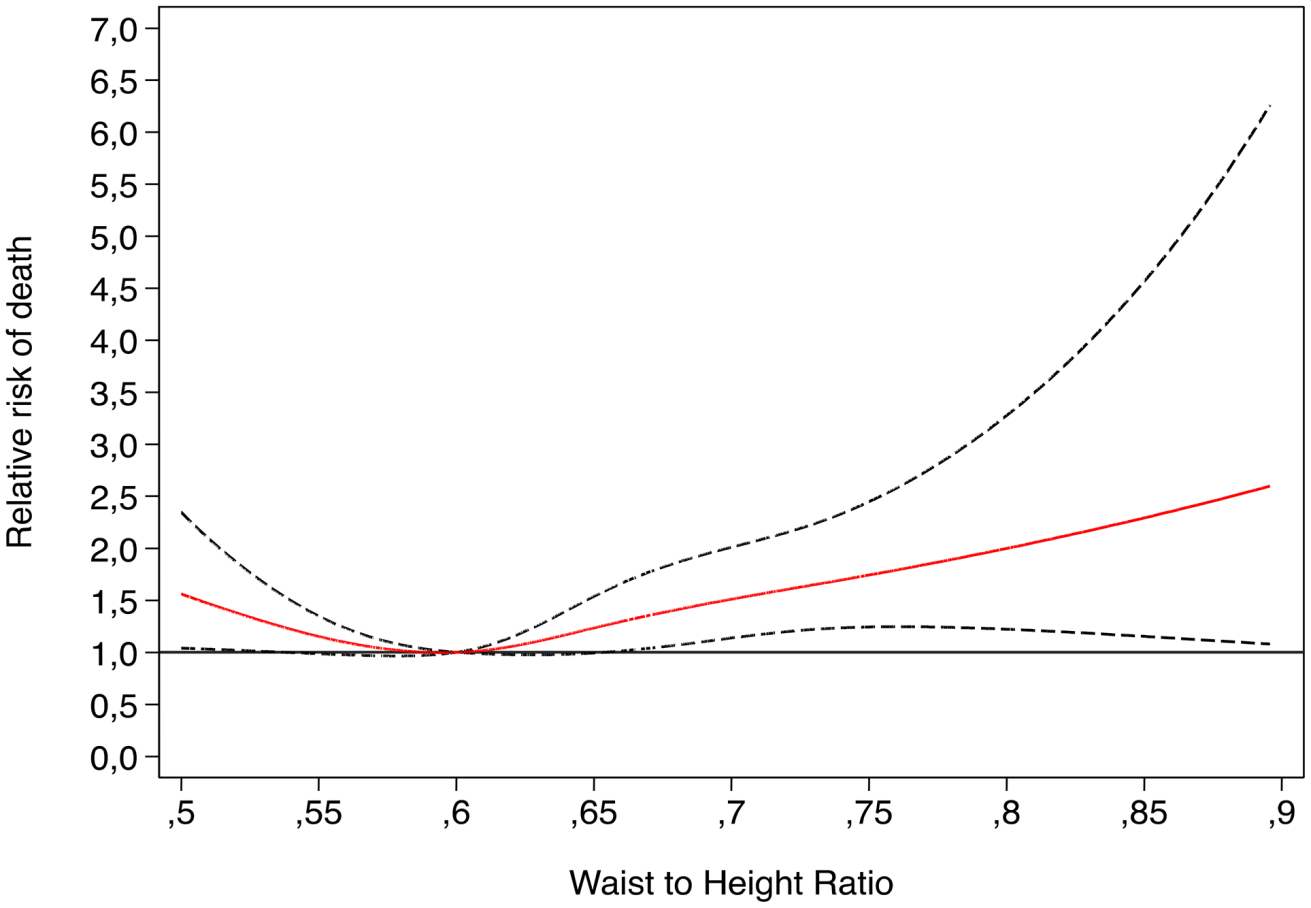
Relative risk of death according to Waist-to-height ratio. Restricted cubic spline model adjusted for age, smoking, diabetes status, hypertensive status, intervention group and family history of CHD.

When we analyzed separately each anthropometric index, we found this same trend. In the case of WHtR (cut-off points: 0.60, 0.65, 0.70) we observed that the hazard ratio monotonically increased. The age and sex-adjusted Hazard ratios (95% confidence intervals) for each category were 1.01 (0.77–1.32), 1.28 (0.96–1.71), and 1.58 (1.11–2.26), for WHtR categories of 0.60–0.65, 0.65–0.70 and >0.70 with respect to <0.65, with a significant direct linear trend (p = 0.009). After multivariable adjustment, these HRs were 1.02 (0.78–1.34), 1.30 (0.97–1.75) and 1.55 (1.06–2.26), respectively with a significant linear trend (p = 0.013). Although the p for interaction with sex was not significant (p = 0.45), when we separated men and women, we observed no significant associations among men after multivariable adjustment. However among women, the multivariable-adjusted HRs for successive categories were 1.34 (0.81–2.22), 1.82 (1.09–3.06) and 1.84 (1.03–3.29). The linear trend among women was significant in both age-adjusted and multivariable-adjusted models (p = 0.036 and 0.016, respectively). This trend was also apparent in the spline analysis (Figure 1).

In the case of WC (cut-off points: 100, 105 and 110 cm) we observed that the hazard ratios increased across successively increasing categories with the exception of one of the intermediate groups (105 to 110 cm). The age-, and sex-adjusted HRs (95% confidence intervals) were 1.14 (0.85–1.53), 1.04 (0.76–1.43) and 1.69 (1.29–2.23) with a significant linear trend (p = 0.002). After multivariable adjustment, these HRs were 1.18 (0.88–1.59), 1.02 (0.74–1.41) and 1.57 (1.19–2.08) with a significant linear trend (p = 0.008). The p for interaction with sex was not significant (p = 0.73). When we divided the sample into men and women, no significant association was observed after multivariable adjustment among men. But the association remained apparent among women (cut-off points: 95,100, 105), with multivariable-adjusted HRs of 1.40 (0.85–2.31), 1.08 (0.60–1.94) and 1.93 (1.21–3.07), with a significant linear trend (p = 0.012).

**Figure 2:**
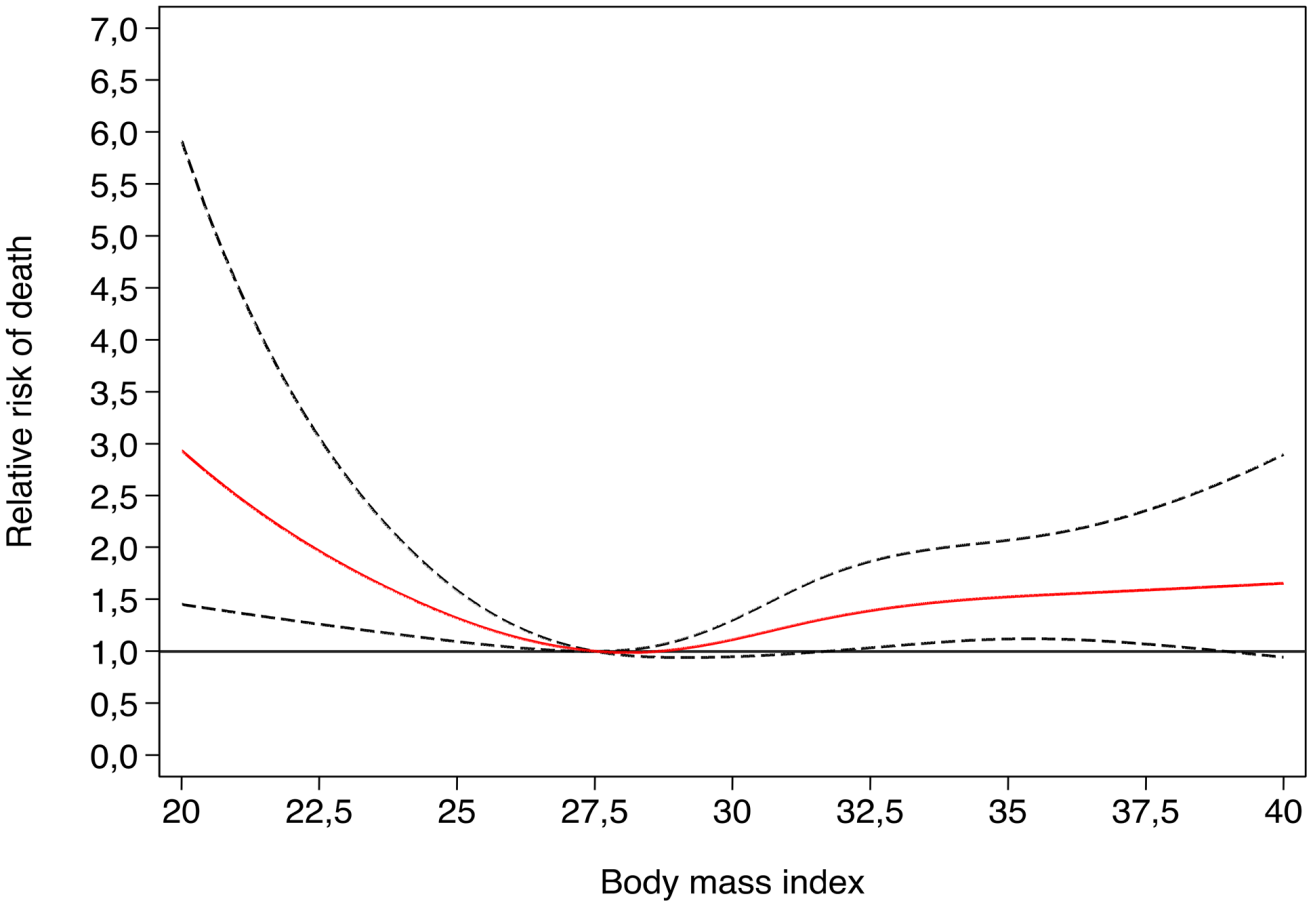
Relative risk of death according to BMI. Restricted cubic spline model adjusted for age, smoking, diabetes status, hypertensive status, intervention group and family history of CHD.

The risk of total mortality across the different categories of BMI (<25, 25–30, 30–35, >35) is shown in the Table 4. We observed that the Hazard ratios decreased in the second group and then they increased in the upper categories. This trend was also apparent in the spline analysis (Figure 2). The p for interaction with sex was not significant (p = 0.43). The adjusted HRs obtained were not significant in men nor women when we stratified the sample by sex. We repeated these analyses after excluding deaths in the first follow-up year and also after excluding ever smokers. In these analyses we found slightly stronger direct associations between BMI and risk of death.

**Table 4:** Hazard Ratios (95% confidence intervals) for total mortality according to categories of body mass index.

	Normal	Overweight	Grade I obesity	Grade II obesity	
**Limits (kg/m^2^)**	**< = 25**	**>25 to 30**	**>30 to 35**	**>35**	
Number of deaths	37	148	135	27	
Person-years	2493	14591	12233	2701	**P for trend**
Age-, sex-adjusted HR	1 (ref.)	**0.74** (0.51–1.06)	**0.91** (0.63–1.31)	**1.06** (0.63–1.78)	0.323
Multivariable adjusted^1^	1 (ref.)	**0.84** (0.58–1.21)	**0.95** (0.66–1.37)	**1.13** (0.66–1.92)	0.387
Excluding deaths in the 2 first follow-up years	1 (ref.)	**1.02** (0.66–1.59)	**1.15** (0.74–1.78)	**1.33** (0.69–2.56)	0.227
Excluding also ever smokers	1 (ref.)	**1.39 (**0.68–2.83)	**1.56 (**0.76–3.17)	**1.76 (**0.70–4.42)	0.175
**Men**					
Number of deaths	26	102	74	11	
Person-years	1092	7096	4816	560	
Age-adjusted HR	1 (ref.)	**0.68** (0.44–1.04)	**0.77** (0.49–1.21)	**1.18** (0.57–2.47)	0.767
Multivariable adjusted^1^	1 (ref.)	**0.70** (0.45–1.10)	**0.71** (0.45–1.12)	**1.48** (0.69–3.19)	0.915
Excluding deaths in the 2 first follow-up years	1 (ref.)	**0.86** (0.50–1.49)	**0.83** (0.47–1.46)	**1.45** (0.50–4.24)	0.958
**Women**					
Number of deaths	11	46	61	16	
Person-years	1402	7494	7417	2141	
Age-adjusted HR	1 (ref.)	**0.83** (0.43–1.59)	**1.11** (0.58–2.12)	**1.12** (0.50–2.49)	0.277
Multivariable adjusted^1^	1 (ref.)	**1.08** (0.52–2.21)	**1.40** (0.70–2.82)	**1.36** (0.57–3.25)	0.199
Excluding deaths in the 2 first follow-up years	1 (ref.)	**1.30** (0.54–3.12)	**1.72** (0.75–3.98)	**1.91** (0.68–5.33)	0.079

The PREDIMED study 2003–2010.

1 Adjusted for age, smoking, diabetes status, hypertensive status, intervention group and family history of CHD.

All estimates are stratified for study center.

When we assessed height, we found increased risk of death in the taller groups with the exception of the second group for men where the HR decreased. The age-sex-adjusted HRs (95% confidence intervals) for each group (cut-off points: 1.65, 1.70, 1.75 cm) were 0.99 (0.77–1.28), 1.10 (0.80–1.52) and 1.57 (1.07–2.31). The linear trend was significant (p = 0.038). But after multivariable adjustment the HRs were not significant. Although the p for interaction was not significant (p = 0.33), when we separated men and women, the age-adjusted HRs were not significant for women (cut-off points: 1.55, 1.60, 1.65). Among men the age -adjusted HRs were 0.96 (0.69–1.34), 1.11 (0.76–1.62) and 1.73 (1.13–2.63), respectively, with a significant linear trend (p = 0.046). However, after multivariable adjustment this association became non-significant.

Almost fifty percent of our participants were diabetics. We investigated the relationship between WHtR and risk of death within the group of diabetic participants (table 5). We found a significant direct linear trend after multivariable adjustment in the diabetic group (p = 0.031). However no statistically significant interaction with diabetes was found (p = 0.85).

**Table 5:** Hazard Ratios (95% confidence intervals) for total mortality according to quartiles of the waist-to-height ratio within diabetics or no diabetics persons.

	1 (lowest)	2	3	4 (highest)	
Limits	0.30 to 0.59	0.59 to 0.63	0.63 to 0.67	0.67 to 1.00	
Number of deaths	83	75	89	101	
Person-years	8188	8132	8059	7642	**P for trend**
Age-, sex-adjusted HR	1 (ref.)	**0.88** (0.64–1.20)	**1.01** (0.75–1.37)	**1.39** (1.02–1.88)	0.027
Multivariable adjusted^1^	1 (ref.)	**0.98** (0.72–1.35)	**1.01** (0.74–1.38)	**1.44**(1.05–1.97)	0.026
**No diabetes**					
Number of deaths	36	31	31	32	
Person-years	4407	4282	3899	3567	
Age-adjusted HR	1 (ref.)	**0.78** (0.49–1.26)	**0.86** (0.53–1.40)	**1.16** (0.70–1.91)	0.587
Multivariable adjusted^1^	1 (ref.)	**0.92** (0.56–1.52)	**0.95** (0.57–1.59)	**1.24** (0.73–2.09)	0.471
**Diabetes**					
Number of deaths	47	44	58	69	
Person-years	3781	3850	4160	4075	
Age-adjusted HR	1 (ref.)	**0.96** (0.63–1.45)	**1.10** (0.75–1.63)	**1.53** (1.04–2.25)	0.021
Multivariable adjusted^1^	1 (ref.)	**1.00** (0.66–1.51)	**1.04** (0.71–1.54)	**1.54** (1.03–2.29)	0.031

The PREDIMED study 2003–2010.

Adjusted for age, sex, smoking, and family history of CHD.

All estimates are stratified for study center. The interaction term was not statistically significant (p = 0.85).

We also studied the relationship between the WHtR index and the risk of all-cause mortality separating the sample according to the randomly allocated group of dietary intervention (arm of the trial) in the Predimed trial: MeDiet with EVOO, MeDiet with nuts, and control diet (Table 6). We found a consistent direct association between WHtR and overall mortality within each of the three intervention arms of the trial. However, the p value for interaction was close to statistical significance (p = 0.056). A significant linear trend after multivariable adjustment suggesting a direct, dose-response relationship between baseline WHtR and mortality was apparent only within the group allocated to a MeDiet with nuts (p = 0.034). In any case, we did not find evidence to support that the PREDIMED intervention was able to counterbalance the harmful effects of increased adiposity on total mortality. Reassuringly, no significant increase in total mortality associated with the active intervention was apparent within the subgroup of participants with the highest levels of WHtR (p = 0.72 for MeDiet + EVOO and p = 0.64 for MeDiet + mixed nuts).

**Table 6:** Hazard Ratios (95% confidence intervals) for total mortality according to quartiles of the waist-to-height ratio within each of the three intervention groups.

	1 (lowest)	2	3	4 (highest)	
Limits	0.30 to 0.59	0.59 to 0.63	0.63 to 0.67	0.67 to 1.00	
**Med Diet with Nuts**					
Number of deaths	26	27	33	30	p for trend
Person-years	2890	2638	2573	2287	
Age-, sex-adjusted HR	1 (ref.)	**1.11** (0.65–1.90)	**1.47** (0.89–2.44)	**1.65** (0.95–2.88)	0.048
Multivariable adjusted^1^	1 (ref.)	**1.19** (0.69–2.07)	**1.53** (0.91–2.57)	**1.76** (0.99–3.11)	0.034
**Med Diet with EVOO**					
Number of deaths	32	27	21	38	
Person-years	2948	3054	3061	2792	
Age-, sex-adjusted HR	1 (ref.)	**0.80** (0.47–1.34)	**0.56** (0.32–0.98)	**1.26** (0.78–2.04)	0.507
Multivariable adjusted^1^	1 (ref.)	**0.86** (0.50–1.46)	**0.54** (0.30–0.98)	**1.25** (0.75–2.08)	0.581
**Control Diet**					
Number of deaths	25	21	35	33	
Person-years	2351	2440	2425	2562	
Age-, sex-adjusted HR	1 (ref.)	**0.79** (0.43–1.43)	**1.23** (0.72–2.09)	**1.27** (0.73–2.01)	0.231
Multivariable adjusted^1^	1 (ref.)	**0.80** (0.44–1.45)	**1.24** (0.73–2.12)	**1.21** (0.68–2.14)	0.311

The PREDIMED study 2003–2010.

1 Adjusted for age, sex, smoking, and family history of CHD.

All estimates are stratified for study center. The interaction term was not statistically significant (p = 0.056).

We fitted restricted cubic spline models to show graphically the relationship between the WHtR (Figure 1) or BMI (Figure 2) and relative risk of death. In figure 1 we present the dose-response relationship between WHtR and total mortality. We found the highest risk of death in participants with the highest levels of abdominal obesity, as measured by the WHtR, and the lowest risk for a WHtR  = 0.6. When we assessed the shape of the dose-response association between BMI and total mortality (Figure 2), we observed that the risk of death was highest for the lowest category of BMI, then it decreased to the lowest risk for values of BMI = 28 kg/m^2^, and then it tended to increase again for upper values of BMI. The comparison of both trends suggested that the upper levels of WHtR are associated with a higher risk of death than the upper levels of BMI.

## Discussion

In this trial of 7447 elderly participants at high cardiovascular risk but initially free of cardiovascular disease, we observed a direct relationship between some adiposity measurements (WC, WHtR, BMI, height) and all cause mortality after 4.8 years of follow-up. However, this relationship was not significant for men and it was not linear in most cases.

A high biological plausibility exists to expect a direct association between excess body weight and all-cause mortality. However, we did not observe any linear trend for body mass index and a curvilinear shaped dose-response association was apparent for indexes of abdominal obesity. Importantly, we observed a lower risk in persons who had moderate levels of overweight compared with normal persons. Thereby, the risk of mortality was sometimes higher for the lowest category of adiposity indexes, specially for BMI, then it decreased and finally it increased again in the upper categories of each anthropometric index. This was in parcial agreement with the results of a recent meta-analysis on BMI and total mortality which concluded that overweight ([BMI] of 25 to 30 kg/m^2^) was associated with significantly lower all-cause mortality in comparison with the normal weight category and that grade I obesity (BMI between 30 and 35) was associated with a non-significantly lower risk of mortality [1]. Other previous metaanalyses also suggested inverse or null associations, specially among elderly subjects. [2], [3]. In any case, methodological aspects deserve caution in the interpretation of some studies included in these meta-analyses. A potential cause of concern is reverse causation bias due to pre-existent disease [24]. In addition, BMI may not be the most appropriate anthropometric index to assess the risks of adiposity.

However, a recent meta-analysis [25] concluded that the existence of a “healthy” pattern of increased weight is not likely to be tenable. On the contrary, any increase in adiposity is associated with increases in levels of cardiovascular risk factors and also in cardiovascular events that will, very probably, lead to increases in total mortality. Excess weight is associated with subclinical metabolic and vascular dysfunction that with the passage of time leads to an increased risk of CV events and mortality. Nevertheless, normal weight persons could have high risk for having metabolic abnormalities as it has been reported recently by De Larochellière et al. [13]. In this context, the routine measurement of WC and the use of WHtR>0.6 as a criterion for excess adiposity would improve the identification of higher-risk subjects. These individuals with normal weight nd increased levels of abdominal obesity could be genetically predisposed to CV disease and could have events even without experiencing increases in body weight or general adiposity. Therefore, it is necessary to consider not only body weight but also metabolic abnormalities. In this context, the use of WC or WHtR should be recommended, instead of using only the BMI.

In this line of thought, one of our contributions in the present work was the use of different indexes of adiposity, and not only BMI. Many previous studies have also used WC. But the use of the WHtR is still scarce. BMI has been widely used by the scientific community to assess obesity. However, BMI cannot distinguish between the accumulation of lean or fat mass, it cannot assess the distribution of fat and the change of this distribution with age nor take into account the changes that occurs in people who begin a diet accompanied or not by exercise.

Interestingly, we found that WC presented a stronger association with mortality than BMI, observing significant results, specially among women. This finding was consistent with other studies [26], [27] showing stronger correlations with visceral fat for WC than for BMI, particulary among elderly participants such as those included in our study.

Recent studies [26], [ 28]–[30] suggest that WHtR is likely to be the best anthropometric index to assess the association of adiposity with cardiovascular risk or overall mortality. This is partially in line with our investigation in which we observed in the restricted cubic spline models that the highest risk of mortality were present in participants from the upper categories of WHtR in contrast with the dose-response trend observed for BMI in which the increased risk of death was less attenuated and the highest relative risk was observed for the leanest subjects. Therefore this is one of the main contributions of our investigation, because we used this anthopometric index (WHtR) and almost no previous studies have longitudinally related this parameter to the risk of death in elderly subjects. In our study we observed that WHtR and WC were the best measures of obesity because using both indexes we obtained significant direct associations, specially for WC. However, WC might underestimate the relative amount of abdominal fat in short persons and overestimate it in tall persons [9]–[11], [15]. This was the reason why we also assessed the role of height. Taking into account that height showed also some associations with mortality, and can introduce confounding in the assessment of the association between WC and mortality, and that the results for WC and WHtR were similar, we consider that WHtR may be preferable and can be used when evaluating CVD mortality risk.

The association between the WHtR and overall mortality was slightly stronger among diabetics. Our study is unique in terms of the large percentage of diabetics (almost fifty percent) of our cohort. However the statistical interaction with diabetes was not significant and further studies on this issue are needed.

Our sample was composed of elderly subjects at high risk of cardiovascular disease, for whom being underweight has been also identified as a predictor of diabetes [31]. However, the precise mechanisms behind the association between low BMI (underweight) and diabetes among the elderly are not yet clarified. A possible explanation could be that protein-calorie malnutrition and magnesium deficiency may cause low insulin secretion and a low pancreatic insuline store. A recent study showed that low dietary magnesium and low mean serum albumin levels were associated with higher risk of type 2 diabetes [32].

However, it is true that abdominal adipose tissue can play an important role in the development of diabetes mellitus and it can also increase the risk of death [33], [34].

Interestingly, after taking into account the dietary intervention conducted in PREDIMED trial, we did not obtain a significant interaction. Therefore we cannot provide evidence that our dietary intervention was able to counterbalance the higher mortality risk associated with higher levels of adiposity. However, in the main results of the PREDIMED trial [17], we found strong evidence for the prevention of major cardiovascular events, but not for total mortality, using a MeDiet supplemented with either extra-virgin olive oil or mixed nuts. A posible explanation could be that when total mortality is used as outcome, we consider all causes of mortality not only cardiovascular causes and there are other important causes of mortality (such as cancer or respiratory causes) where subclinical disease is associated with weight loss. Another aspect to be considered is that in the PREDIMED trial the average WC of participants was greater than 100 cm and the average BMI was greater than 30 kg/m^2^. In addition, these participants were at high cardiovascular risk because they had other cardiovascular risk factors and fifty percent of them were type-2 diabetics. Nevertheless the protocol of the PREDIMED intervention did not include specific caloric restriction or targeted goals for weight loss. In this context, and given the present results, a trial using an energy-restricted MeDiet would be likely to obtain even greater cardiovascular benefits. The new on-going trial referred to as PREDIMEDPLUS (www.predimedplus.com) will assess the effect of this combined intervention on major cardiovascular events and total mortality.

There are several strengths in our research. The prospective nature of our study with a long follow-up period allowed us to detect a sufficient number of deaths. In addition we were able to control for multiple potential confounders using multiple-adjusted models. The results could suggest causal associations because in Cox regression we controlled for a high number of potential confounders. However, residual confounding by unmeasured factors is always a possibility in observational research.

In contrast, the older age of our participants together with their high cardiovascular risk could contribute to modify the association between adiposity measures and all-cause mortality. Obesity has been associated with improved survival in patients with existing chronic diseases, including chronic obstructive pulmonary disease, chronic kidney disease, or heart failure. This fact has been termed the “obesity paradox” [24], [35]. This paradox may attenuate the detrimental effects of adiposity on mortality. In addition, total body mass increases across the life span until about age 60 and then it tends to decrease due to the process of sarcopenia which involves a decrease in muscle mass and on the other hand a smaller increase in fat mass. Moreover, at that age a fat re-distribution occurs, and, as a result of this re-distribution, the fat that was out of the trunk tend to be displaced to abdominal areas, thus increasing the metabolic risk [36]. Previous studies have suggested that BMI do not capture the increase in abdominal adipose tissue that tends to occur with age and this could explain the weakened association between BMI and risk of death that is usually observed with age [29]. All these reasons support the preferential use of the WHtR in elderly subjects.

Previous studies suggested a weak relationship of WC and BMI with cardiovascular risk in older adults [3], [37], [38]. Stevens et al. [7] described that the relative risk of all-cause and cardiovascular mortality associated with an excess body mass is greater for younger and middle-aged individuals than for older individuals. However, this effect modification by age has been recently challenged because the reduction in the magnitude of the association obesity–mortality with age maybe confounded by age at survey and cohort effects [39].

For these reasons, the present research gained more importance. Notwithstanding that in our study all participants were elderly subjects, we found a significant direct relationship between some adiposity measures (specially WC and WHtR) and all-cause mortality. But as in other studies, we found a weaker relationship for BMI than for these other measures.

A possible explanation for the non-significant relationships or the weaker associations found among men than among women, is the well-known longer survival of women. There could be a selective survival effect of healthier, older men because the unhealthy men with higher levels of adiposity at high cardiovascular risk may have already died from the detrimental effects of obesity. In addition, older individuals tend to decrease their intake of food and may not consume energy-rich diets that are related to a higher risk of death.

In conclusion, our study showed a direct association between adiposity measures reflecting visceral accumulation of fat (WC, WHtR) and all-cause mortality in an elderly population at high cardiovascular risk. The highest risk of death was observed in participants who initally were in the highest quartiles of these anthropometric indexes (WC, WHtR) that better capture abdominal obesity. For BMI, instead, we observed an U-shaped dose-response pattern, with the highest mortality in participants with the initial lowest values of BMI. However, further research is warranted to confirm these findings and to extend our findings to other elderly populations with a lower cardiovascular risk.
